# Supplementary material for: Chromosome-Scale Genome Assembly and Triterpenoid Saponin Biosynthesis in Korean Bellflower (Platycodon grandiflorum)
Source: Int J Mol Sci. 2023 Mar 31;24(7):6534. doi: 10.3390/ijms24076534 (PMC10095269; doi:10.3390/ijms24076534)
Supplement: Supplementary file 1 [file ijms-24-06534-s001.zip › Supp_Figures.pdf]

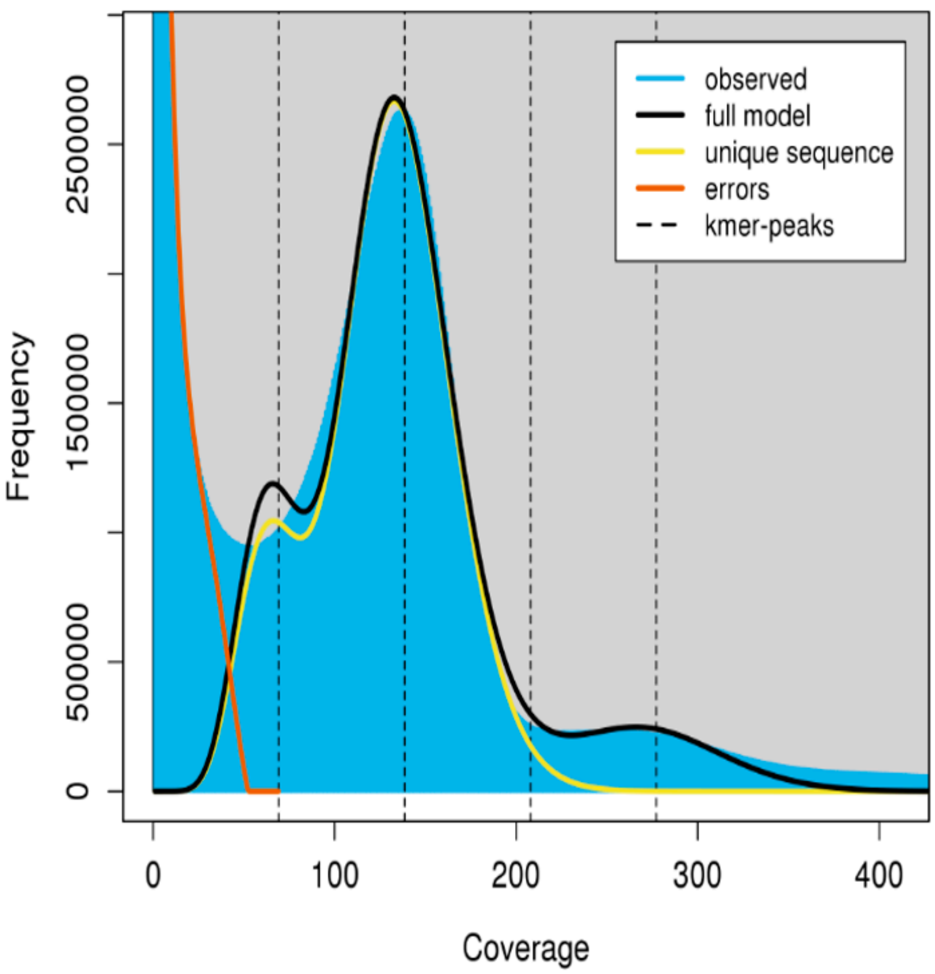

Supplementary Figure S1. Genome size estimation of *P. grandiflorum* by profile plots which of *k*-mer frequency at a *k*-mer length of 17. These plots were drawn using GenomeScope (version 2.0) with *k*-mer frequency data which obtained from high-quality Illumina PE data.

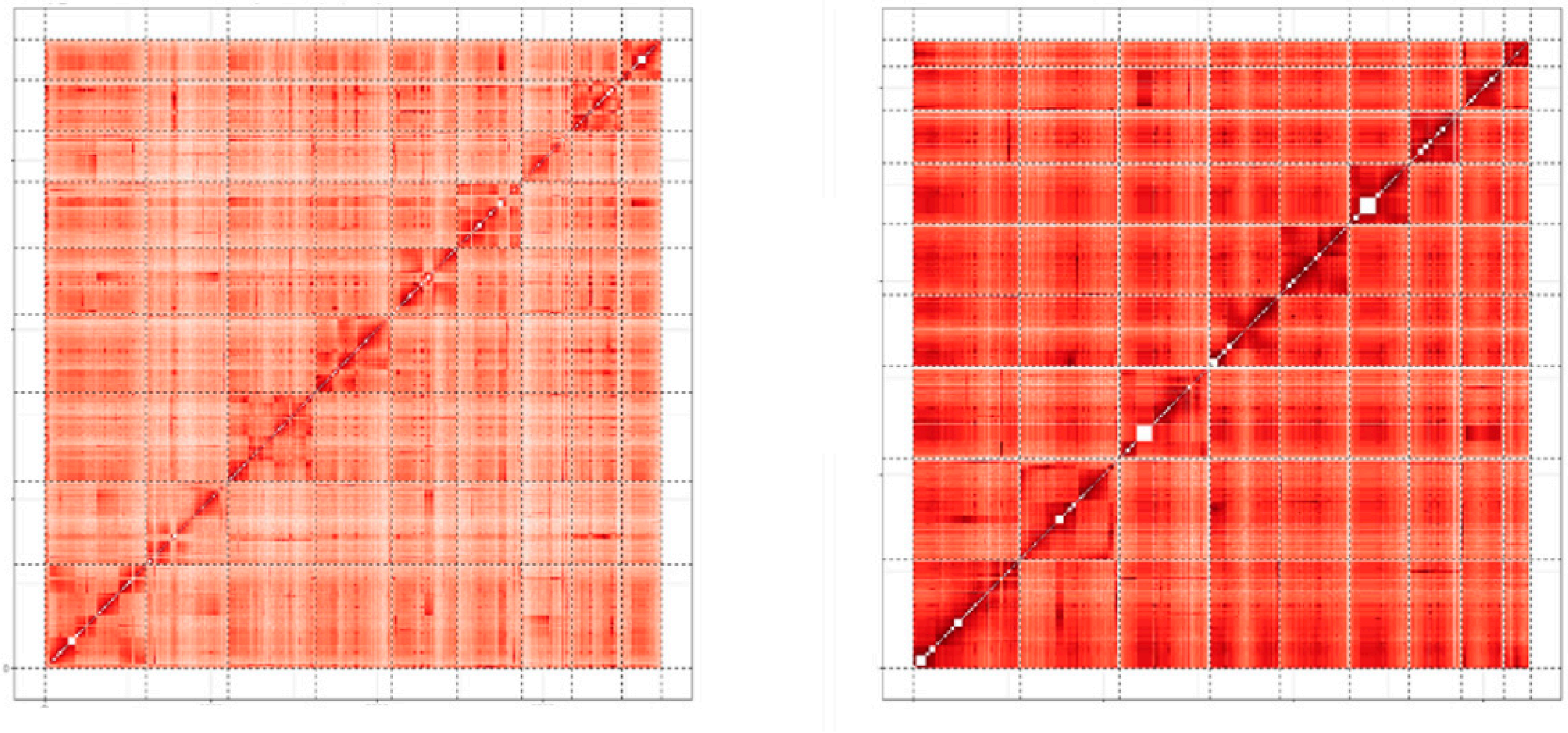

<ONT_SMARTdn> <ONT_Canu>

Supplementary Figure S2. Hi-C interaction heatmap generated by preliminary Hi-C analyses for two contig sets assembled by SMARTdenovo (ONT_SMARTdn) and Canu (ONT_Canu) assembler tool, respectively.

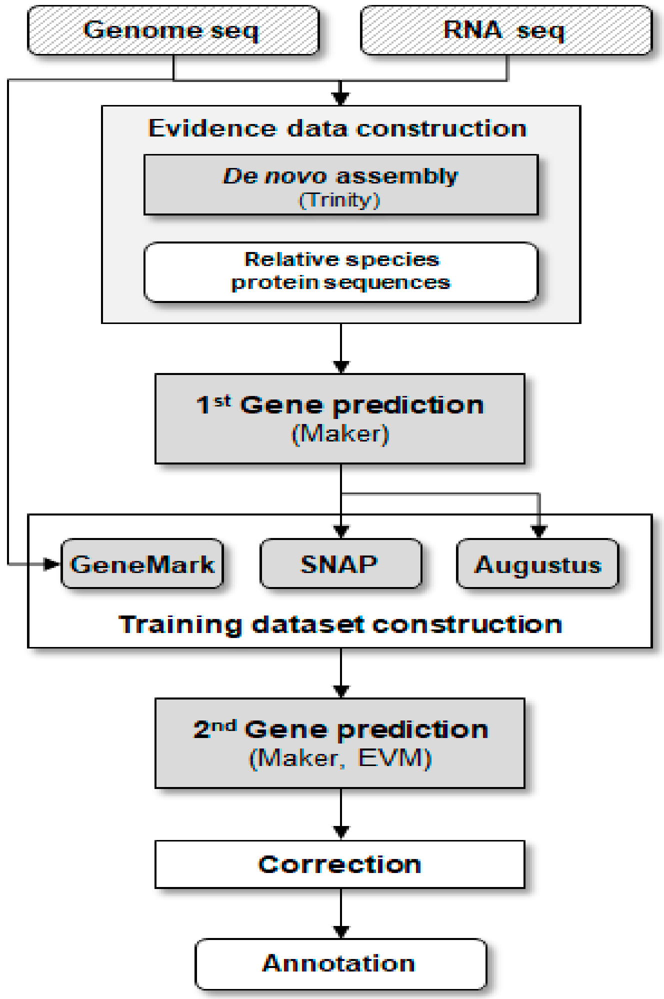

Supplementary Figure S3. Annotation pipeline for gene prediction from the genome sequence of *P. grandiflorum*. Multiple annotation tools used are Trinity (Grabherr et al., 2011), MAKER3 (https://www.yandell-lab.org/), SNAP (Zaharia et al., 2011), AUGUSTUS (Stanke et al., 2006), GeneMark-ES (version 4.38, Alexandre et al., 2005) and EvidenceModeler (version 1.1.1, Haas et al., 2008).

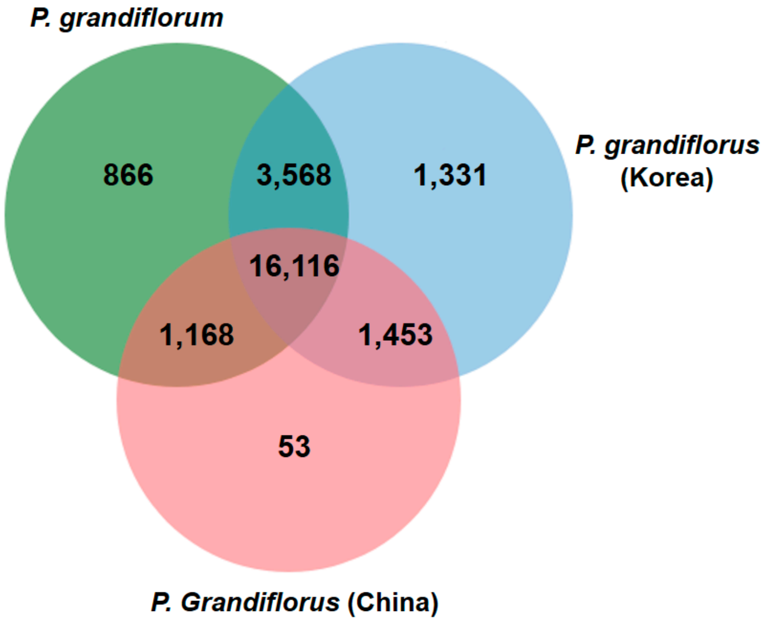

Supplementary Figure S4. Shared and unique gene clusters among *P. grandiflorum and* two *P. grandiflorus genomes.* Two Platycodon grandiflorus species indicate the Korean (Kim *et al.,* 2020) and Chinese balloon flower (Jia *et al.,* 2022). Venn diagram was drawn using the OrthoVenn2 web tool.

.
